# The *Aspergillus niger *multicopper oxidase family: analysis and overexpression of laccase-like encoding genes

**DOI:** 10.1186/1475-2859-10-78

**Published:** 2011-10-08

**Authors:** Juan A Tamayo Ramos, Sharief Barends, Raymond MD Verhaert, Leo H de Graaff

**Affiliations:** 1 Fungal Systems Biology, Laboratory of Systems and Synthetic Biology, Wageningen University, Dreijenplein 10, 6703 HB, Wageningen, The Netherlands; 2 ProteoNic BV, Niels Bohrweg 11-13, 2333CA Leiden, The Netherlands; 3 Genencor International, 925 Page Mill Road, Palo Alto, CA 94304, USA

**Keywords:** Multicopper oxidase, laccase, *Aspergillus niger*, secretion

## Abstract

**Background:**

Many filamentous fungal genomes contain complex groups of multicopper oxidase (MCO) coding genes that makes them a good source for new laccases with potential biotechnological interest. A bioinformatics analysis of the *Aspergillus niger *ATCC 1015 genome resulted in the identification of thirteen MCO genes. Ten of them were cloned and homologously overexpressed.

**Results:**

A bioinformatic analysis of the *A. niger *ATCC 1015 genome revealed the presence of 13 MCO genes belonging to three different subfamilies on the basis of their phylogenetic relationships: ascomycete laccases, fungal pigment MCOs and fungal ferroxidases. According to *in silico *amino acid sequence analysis, the putative genes encoding for functional extracellular laccases (*mcoA*, *mcoB*, *mcoC*, *mcoD*, *mcoE*, *mcoF*, *mcoG*, *mcoI*, *mcoJ *and *mcoM*) were placed under the control of the *glaA *promoter and overexpressed in *A. niger *N593. Enzyme activity plate assays with several common laccase substrates showed that all genes are actually expressed and code for active MCOs. Interestingly, expressed enzymes show different substrate specificities. In addition, optimization of fungal pigment MCOs extracellular production was investigated. The performance of the widely used glucoamylase signal sequence (ssGlaA) in McoA secretion was studied. Results obtained suggest that ssGlaA do not yield higher levels of secreted McoA when compared to its native secretion signal. Also, McoB synthesis was investigated using different nitrogen sources in minimal medium liquid cultures. Higher yields of extracellular McoB were achieved with (NH_4_)_2 _tartrate.

**Conclusions:**

*Aspergillus niger *is a good source of new laccases. The different substrate specificity observed in plate assays makes them interesting to be purified and biochemically compared. The homologous signal sequence of McoA has been shown to be a good choice for its extracellular overexpression. From the nitrogen sources tested (NH_4_)_2 _tartrate has been found to be the most appropriate for McoB production in *A. niger*.

## Background

Multicopper oxidases (MCOs) form a family of enzymes that is widely distributed in nature, including laccases (EC 1.10.3.2), ascorbate oxidases (EC 1.10.3.3), bilirubin oxidases (EC 1.3.3.5) and ferroxidases (EC 1.16.3.1) [1,2]. MCOs catalyze the oxidation of a variety of substrates (mainly aromatic compounds and metals) concomitantly with the reduction of molecular oxygen to water. Their ability to catalyze reactions by producing just water as the only by-product has increased the interest of the industry for their use as 'green' catalysts, and their importance is reflected in the broad spectrum of applications, that range from pulp delignification, textile dye bleaching and water or soil detoxification, to the formation of pigments, the development of clinical tests and applications in the field of biosensors, bioreactors, and biofuel cells [2,3]. Laccases form the biggest subgroup within the MCO family, and due to their broad substrate specificity they attract the most attention in studies for biotechnological applications, although other family members like ascorbate oxidases and bilirubin oxidases have shown to be promising as biocatalysts for the industry as well [2,3]. Fungal pigment MCOs are able to oxidize typical laccase substrates as *p*-phenylenediamines, pyrogallol, gallic acid or 2,2-azino-di(3-ethylbenzthiazoline) sulfonic acid (ABTS) [4,5], but no studies about their possible industrial applications have been reported.

MCO coding genes appear to be redundant in fungal genomes, probably due to the fact that their coding products may play different physiological roles and they can be differently regulated depending on environmental conditions [3,6]. This phenomenon has been described mostly in basidiomycetes like *Coprinopsis cinerea*, that contains 17 laccase *sensu stricto *coding genes [7] and *Laccaria bicolor *with 9 laccase *sensu stricto *and 2 ferroxidase genes [8]. In ascomycetes MCOs have been much less studied [9]. *Aspergillus nidulans *produces YA [10] and LccD [5], belonging to the fungal pigment MCO subgroup and the laccases *sensu stricto *LccB and LccC [5]. Distinct MCO genes and their coding products have been also characterized in *Neurospora crassa*, *Aspergillus fumigatus*, *Myceliophthora thermophila*, *Melanocarpus albomyces*, and more recently in *Trichoderma reesei *[11-15]. The production of active laccases has been reported in many other ascomycetes [16], but no complete families in fungi from this phylum have been described so far. Therefore, it is difficult to have an actual overview about the biotechnological potential of ascomycete genomes as source for new MCOs. Regarding *Aspergillus niger*, widely used as an amenable tool for laccase production together with other cell factories like *Pichia pastoris*, *T. reesei *or *Aspergillus oryzae *[17-19], it is remarkable that limited information is available about its potential as multicopper oxidases source, whereas various oxidoreductases, including a laccase from black aspergilli, are already being industrially commercialized (http://www.amfep.org/documents/Amfep List of Commercial Enzymes - OCT2009 - Amfep 09 68.pdf). Only recently, BrnA, a MCO involved in the conidial pigmentation of *A. niger *has been described [20]. The fact that a large number of three-dimensional structures of basidiomycete laccases have been published, whereas only two 3D structures from the ascomycete laccases *sensu stricto *of *Melanocarpus albomyces *[21] and *Thielavia arenaria *[9] have been reported, and no one is available from the fungal pigment MCO subfamily, also indicates the lack of information generated from ascomycete MCOs, particularly from the latter group, when compared to basidiomycetes.

In this investigation ten *A. niger *MCO putative genes were selected on the basis of their phylogenetic relationships with other multicopper oxidases with reported laccase activity and homologously expressed from the *glaA *promoter. Optimization of the extracellular levels of fungal pigment MCOs was investigated using different approaches.

## Results and discussion

### Identification and phylogenetic characterization of *A. niger *MCOs

**Figure 1:**
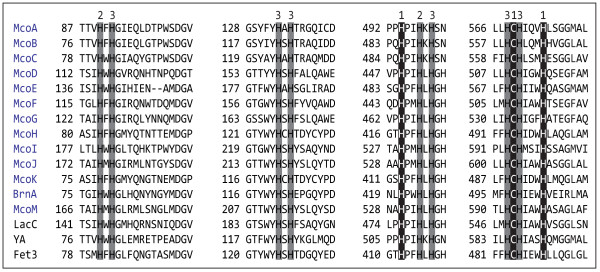
**Sequence alignment of the four copper-binding sites in the 13 *A. niger *ATCC 1015 MCOs with *N. crassa *ascomycete laccase (LacC), *S. cerevisiae *ferroxidase (Fet3) and *A. nidulans *fungal pigment MCO (YA)**. Numbers indicate the histidine (H) and cysteine (C) copper ligands, according to the type of Cu they bind [56].

A BLASTp search was made against the *A. niger *ATCC 1015 genome to identify its complete MCO coding gene family. The sequences of 38 characterized multicopper oxidases, from different origin and with different biological roles, were used as query. As a result, 13 gene models containing the residues proposed to be involved in copper coordination (Figure 1) --a characteristic signature of all MCOs-- were identified (Table 1), including the recently characterized BrnA [20]. Sequence identities among the 13 deduced proteins ranged from 21 to 69% with McoE and McoI having the lowest identity to the others (35% ≤). McoA, McoB and McoC where at least 52% identical among them, whereas McoD, McoF and McoG were no less than 50% identical between themselves. In addition McoH and McoK showed the highest degree of identity (69%). Signal P 3.0 predicted a signal peptide for all the deduced proteins with the exception of McoI, suggesting therefore an intracellular localization for the latter. However the SecretomeP 2.0 Server, a tool designed to predict whether a protein is secreted via non-classical pathways that has been used repeatedly in the analysis of fungal sequences [22-25], predicted that McoI might be an extracellular protein. The TMHMM Server 2.0 predicted a transmembrane domain in the C-terminal region of McoH and McoK.

**Table 1:** Protein accession number, predicted nucleotide and amino acid sequence length of the *A. niger *ATCC 1015 and CBS 513.88 strains MCOs

Gene	Accession n° ATCC 1015	Accession n° CBS 513.88	Nucleotide sequence length (bp)	Deduced primary polypeptide length (aa)
*mcoA*	e_gw1_1.1387	An01g14010	1907	602

*mcoB*	e_gw1_1.1368	An01g13660	1922	596

*mcoC*	fge1_pg_C_14000044	An03g03750	1979	614

*mcoD*	e_gw1_4.1637	An11g03580	1806	563

*mcoE*	fge1_pg_C_1000314	An01g11120	2143	586

*mcoF*	fge1 e_gw1_12.409	An05g02340	1876	559

*mcoG*	fge1_pm_C_3000179	An08g08450	1840	594

*mcoH*	e_gw1_1.309	An01g08960	1842	613

*mcoI*	gw1_10.607	An18g02690	2188	648

*mcoJ*	gw1_9.210	An12g05810	2491	672

*mcoK*	fge1_pg_C_6000422	An15g05520	1845	614

*brnA*	e_gw1_8.661	An14g05370	1722	547

*mcoM*	gw1_7.235	An16g02020	2413	658

*mcoN*	-	An01g00860	1856	598

*mcoO*	-	An04g10400	1946	605

*mcoP*	-	An05g02540	1903	588

**Figure 2:**
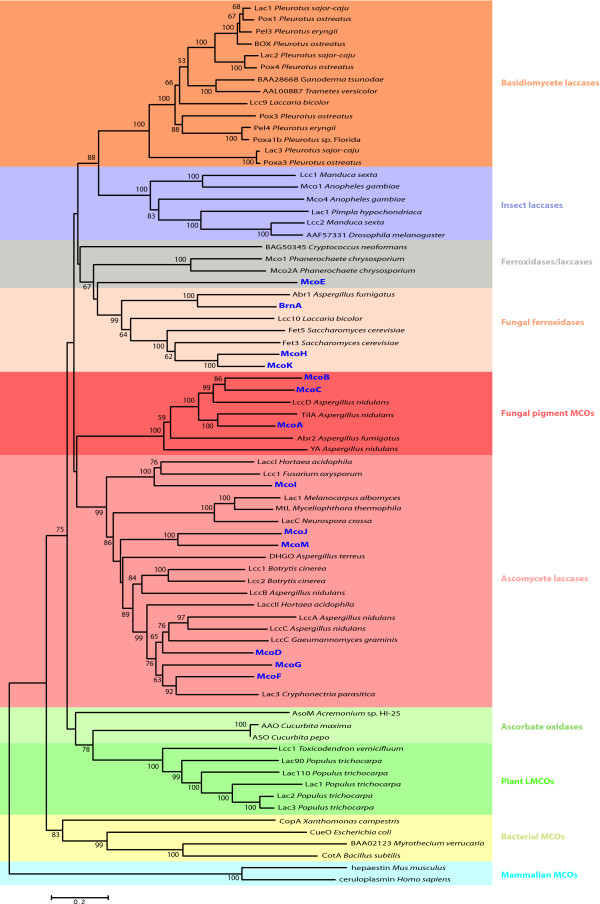
**Neighbor joining tree of multicopper oxidase amino acid sequences**. The *A. niger *MCOs (in dark blue) are distributed amongst the ascomycete laccases, fungal pigment MCOs, fungal ferroxidases and ferroxidases/laccases subfamilies. The phylogenetic tree was constructed with MEGA4, using the Poisson correction model, pairwise deletion and 500 replicates. Only values ≥ 50% are shown.

In order to identify those *A. niger *MCOs that are more likely to have laccase activity, a phylogenetic analysis was performed to investigate their relationships with a number of already characterized multicopper oxidases from different origins (Figure 2).

The products of genes *mcoA*, *mcoB *and *mcoC *cluster with fungal pigment MCOs. This group contains the enzymes YA from *A. nidulans *and Abr2p from *A. fumigatus*, both involved in conidial pigment biosynthesis and reported to have laccase activity, and TilA, which is localized at the growing hyphal tip in *A. nidulans *and has unknown function [10,15,26].

McoD, McoF, McoG, McoI, McoJ and McoM are in the cluster that groups the ascomycetous laccases (*sensu stricto*) that contains characterized laccases from *Botrytis cinerea *[27], *N. crassa *[14], *M. albomyces *[13] and *A. nidulans *[5] among others.

The MCOs McoH, McoK *and *BrnA cluster in the fungal ferroxidase family, that includes the Fet3 and Fet5 proteins, responsible for the reductive iron assimilation system in *Saccharomyces cerevisiae *[28,29]. This cluster also harbors the vermelone dehydratase Abr1, involved (together with Abr2) in the biosynthetic pathway of melanin in *A. fumigatus *[30,31]. McoH and McoK are both 49% identical to Fet3 and their coding genes are clustered with putative iron permease genes (*ftrA *and *ftrB*) whose coding products are 51% and 48% identical to *S. cerevisiae *Ftr1 respectively. Fet3 and Ftr1 are responsible of the high affinity iron transport in *S. cerevisiae*, therefore a similar function is predicted for McoH and McoK in *A. niger*.

McoE belongs to the ferroxidases/laccases subgroup, a grade of basidiomycete and ascomycete MCOs that includes Mco1 from *Phanerochaete chrysosporium *and a laccase from *Cryptococcus neoformans*, both of them with strong ferroxidase and weak laccase activities [1].

The number of MCO coding genes present in *A. niger *ATCC 1015 is the highest reported so far in any ascomycete, and higher than those reported in basidiomycetes, with the exception of *Coprinopsis cinerea*, whose genome contains 17 MCO coding genes [7,8]. Also, significant genotypic differences have been recently found between *A. niger *strains [32]; the genome mining of the CBS 513.88 strain revealed the presence of 16 MCO genes (Table 1). Of these, 13 homologous to the ones present in ATCC 1015 and three additional full-length coding genes: the ascomycete laccase McoN, similar to McoI (58% identical) and the fungal pigment MCOs McoO and McoP, similar to McoA (56% identical) and McoC (57% identitical) respectively, highlighting even more the potential of *A. niger *as an interesting source for new multicopper oxidases.

### Homologous overexpression of *A. niger *MCO coding genes with possible biotechnological interest

The analysis of the amino acid sequences and the phylogenetic relationships of the putative *A. niger *ATCC 1015 MCOs indicates that at least 9 of the 13 coding genes could code for extracellular enzymes with laccase activity. As mentioned before, laccases have been reported both in fungal pigment MCO and ascomycete laccase *sensu stricto *subgroups, thus all *A. niger *MCO genes included in the two subfamilies and predicted to be extracellular were selected for their homologous overexpression. The *mcoI *gene was also selected as it could be targeted to a non-classical secretory pathway and its hypothetical coding product is 49% identical to the laccase I of *Hortaea acidophila *[33]. In addition *mcoE *from the ferroxidases/laccases grade was selected, as its coding product is predicted to be an extracellular enzyme and no similar MCO has been characterized so far. McoH and McoK have been predicted to be iron transporters located in the plasma membrane, therefore both of them were discarded, whereas BrnA was not selected, as no laccase activity has been reported for it and its ortolog Abr1 in *A. fumigatus *[20,30,31].

*mcoA*-G, *mcoI*, *mcoJ *and *mcoM *were homologously expressed in the *A. niger *N593 strain under the control of the widely used *glaA*-promoter (*glaA*_p_) [34]. Due to probable ectopic integrations of the plasmids carrying the MCO genes, different expression efficiencies can occur. Therefore at least 15 transformants expressing each gene were tested in plate activity assays for ABTS and 4-amino-2,6-dibromophenol/3,5-dimethylaniline (ADBP/DMA) conversion. A significant number of transformants per gene showed laccase activity against one or both substrates. As expected, differences were observed in expression levels for each group of transformants. The additional file 1 in the supplementary material illustrates the screening for McoJ positive transformants (around 60%) in an ADBP/DMA plate assay. Different expression levels can be observed: from no detectable activity to a maximum rate of ADBP/DMA conversion, reached by several transformants.

**Figure 3:**
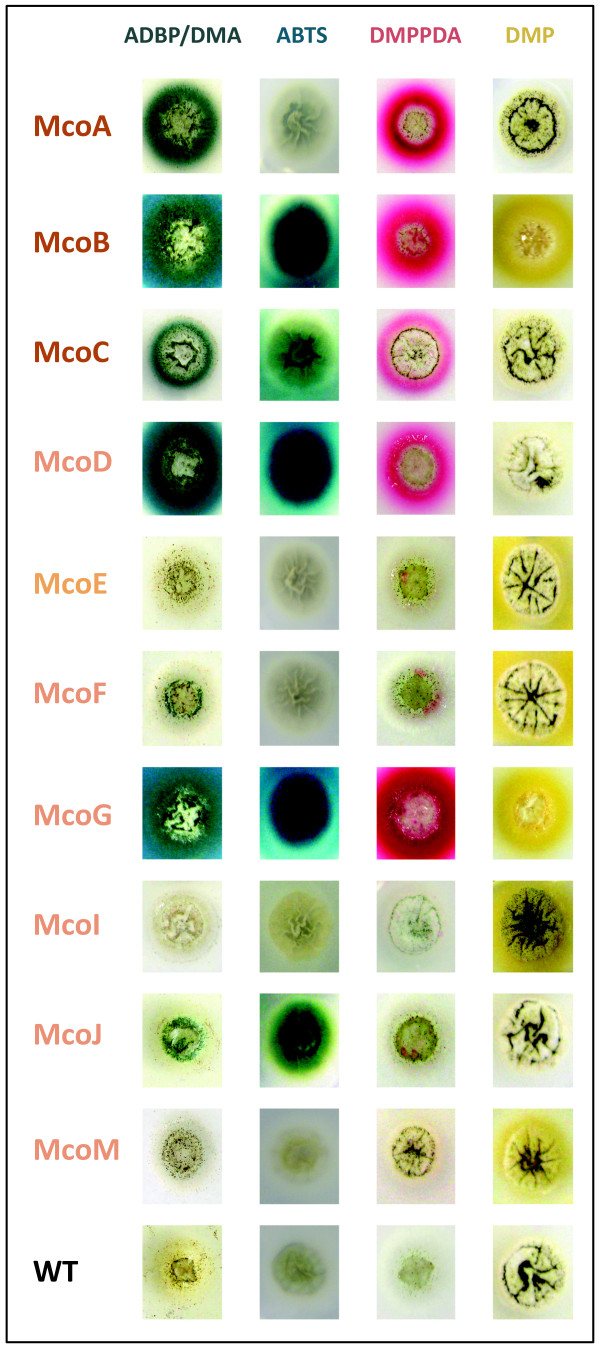
**Enzyme activity plate assays of *A. niger *N593 strain expressing McoA, McoB, McoC, McoD, McoE, McoF, McoG, McoI, McoJ and McoM in the presence of substrates ADBP/DMA, ABTS, DMPPDA and DMP**. WT refers to *A. niger *N593 transformed with the empty vector pALIV.

When ADBP/DMA was used as a substrate laccase activity was detected in a significant number of transformants expressing McoA, McoB, McoC, McoD, McoF, McoG and McoJ. Only strains expressing McoB, McoC, McoD, McoG and McoJ showed laccase activity towards ABTS. Clear differences in substrate specificity were also reported for *A. nidulans *laccases when ADBP/DMA and ABTS were used in plate assays [5]. Therefore, in order to have more insight in *A. niger *MCOs substrate specificity two more laccase substrates - N, N-dimethyl-*p*-phenylenediamine sulfate (DMPPDA) and 2,6-dimethoxyphenol (DMP) - were tested in plate assays. Again, at least 15 transformants expressing McoE, McoI and McoM were tested, whereas for the other MCOs (McoA, McoB, McoC, McoD, McoF, McoG and McoJ), only the transformant with the higher activity towards the substrates used previously was tested in parallel. For these substrates also different patterns of enzyme activity were observed: only McoA, McoB, McoC, McoD and McoG reacted with DMPPDA, whereas McoB, McoE, McoF, McoG, McoI and McoM transformants showed activity when assayed with DMP. Different degrees of specificity could be observed too depending on the color intensity of the reaction product: green-blue halo for ADBP/DMA, green halo with ABTS oxidation, pink halo for DMPPDA and yellow halo in the case of DMP (Figure 3).

The substrate specificity of *A. niger *MCOs appeared not to be correlated to their primary structures, as both ascomycete laccase *sensu stricto *and fungal pigment MCO subgroups include enzymes that were able to oxidize the four substrates: McoB and McoG; enzymes that clearly reacted with all of them but DMP: McoC and McoD; and enzymes that only showed activity with two substrates: McoA (ADBP/DMA and DMPPDA), McoF (ADBP/DMA and DMP) and McoJ (ADBP/DMA and ABTS). McoE, McoI and McoM were only able to oxidize DMP and very weakly, as the oxidation of the substrate was only visible after four days. However, as reported for other fungal MCOs, they may need other conditions like: a different concentration of CuSO_4_, substrate, incubation temperature and/or pH to perform their activity optimally [16,35].

The patterns of activity observed in plate assays for each *A. niger *MCO against the different substrates suggest that remarkable biochemical differences exist between them. Therefore, further studies should be done with respect to their biochemical characterization and their possible biotechnological potential.

### Optimization of fungal pigment MCOs extracellular production

Due to the wide range of possible applications of multicopper oxidases many studies are focusing on the improvement of their production levels and their molecular characterization [36]. Fungal pigment MCOs are still a far less explored group of copper-containing polyphenol oxidases in comparison to laccases *sensu stricto*. Although some of them have been homologous expressed in *A. nidulans*, being used as a reporter system [5], no overproduction studies have been described. In that sense, the performance of the GlaA signal sequence (ssGlaA), successfully used to improve the secretion of many proteins, including recombinant laccases [37-39], as well as different culture conditions, were analyzed in this work.

To investigate the effect of ssGlaA in the production of extracellular fungal pigment MCOs McoA was chosen as a model enzyme. *A. niger *transformants ectopically expressing McoA with ssGlaA instead of their native signal sequence (ssMcoA) were generated, and their performance compared with McoA producers with ssMcoA.

**Figure 4:**
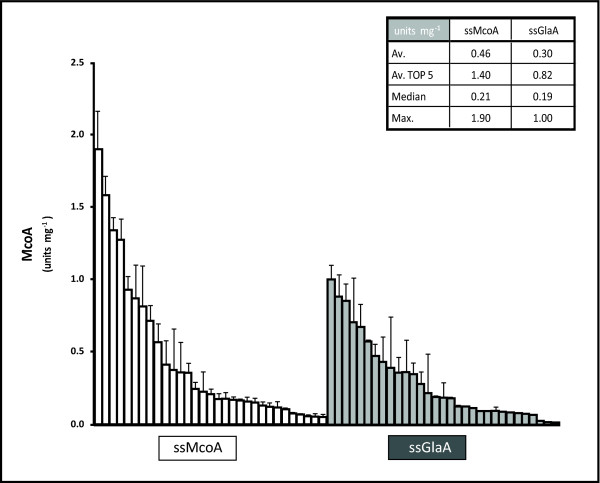
**Laccase activity (units mg^-1 ^total secreted protein) in culture supernatants of *A. niger *N593 transformants expressing McoA with its native secretion signal (ssMcoA) (white bars) and with ssMcoA replaced by the glucoamylase leader sequence (ssGlaA) (grey bars)**. The embedded table shows the average (Av.), the average of the five best producers (Av. TOP 5), the median and the higher levels (Max.) of McoA secreted by each group of transformants.

Thirty transformants per construct were grown in 96 well culture plates using minimal medium with 50 mM maltose and 0.1 mM CuSO_4_. After 24 hours McoA production was quantified using ADBP/DMA as a substrate. Figure 4 shows the McoA activity of the sixty transformants, expressed in units mg^-1 ^total protein secreted. The strains based on the ssMcoA resulted to produce on average higher levels of extracellular McoA in comparison to ssGlaA strains. The best producer carrying ssMcoA was able to produce 90% more enzyme than the best one with ssGlaA, whereas the difference in McoA production between the top five producers from both conditions was around 70%. This result indicates that GlaA signal sequence is not more efficient than ssMcoA in the production of extracellular McoA. However, further experiments should be performed to elucidate whether ssGlaA produces a positive effect in McoB or McoC extracellular levels.

**Figure 5:**
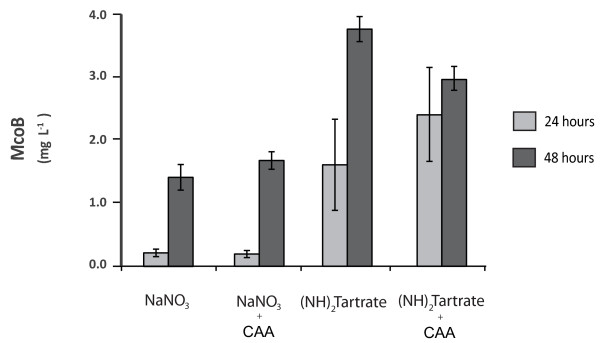
**McoB (mg L^-1^) in supernatants of cultures containing different nitrogen sources at 24 (clear grey bars) and 48 (dark grey bars) hours after inoculation**. The concentration of NaNO_3 _and (NH_4_)_2 _tartrate was 70 mM. CAA as a supplement was used in a final concentration of 0.1%.

To investigate the effect of different culture conditions McoB was used as a reporter. As the effect of a number of carbon sources in the performance of the GlaA expression system has been recently deeply investigated [40], various organic and inorganic nitrogen sources were tested in this work. The best McoB producer out of 15 strains tested in plate assays was grown in 100 mL of minimal medium with the following nitrogen sources: urea, NH_4_CL, casamino acids (CAA), NaNO_3 _and (NH_4_)_2 _tartrate. Culture supernatant samples were taken 24 and 48 hours after inoculation and McoB production was quantified using DMPPDA as a substrate. Only detectable amounts of McoB were measured when NaNO_3 _and (NH_4_)_2 _tartrate were present in the media. Additionally, McoB production in cultures with NaNO_3 _and (NH_4_)_2 _tartrate supplemented with 0.1% of CAA were also tested (Figure 5). As shown in Figure 5, cultures with (NH_4_)_2 _tartrate yielded higher levels of McoB, independently of the sampling point or the absence/presence of CAA. At 24 hours after inoculation McoB activity was 8 times higher in (NH_4_)_2 _tartrate cultures when no CAA were added and 13 times higher with CAA. At 48 hours McoB activity was still 2.7 and 1.8 times higher when (NH_4_)_2 _tartrate was used as a substrate, in the absence or presence of CAA respectively. Therefore (NH_4_)_2 _tartrate is the best choice for McoB production over all nitrogen sources and conditions tested in this study. Also, in contrast to the positive effects reported in the *A. niger *GlaA expression when the culture medium was supplemented with 0.5% CAA [41], no clear positive effect was observed in McoB production with a supplement of 0.1% of CAA. On the other hand, although no high titres of McoB were achieved in the culture conditions studied, a further screening of transformants has allowed the isolation of strains able to produce around 10 times higher levels to the ones presented in Figure 5 (Tamayo-Ramos *et al*., manuscript in preparation). The use of strategies based on efforts done so far to boost yields of recombinant proteins (in particular laccases) [40-44], should result in new increases of McoB production levels.

## Conclusions

The complete family of *A. niger *MCO coding genes was identified in the acidogenic wild-type strain ATCC 1015. Those genes coding for putative extracellular laccase-like MCOs were selected after the analysis of their theoretical coding products primary structure and phylogenetic relationships. After their cloning and overexpression in *A. niger *different plate enzyme activity assays showed that all genes code for active multicopper oxidases, with apparent different degrees of affinity and specificity. The use of the *A. niger *GlaA secretion signal did not suppose an increase in McoA extracellular production and (NH_4_)_2 _tartrate resulted to be the most appropriated nitrogen source for McoB synthesis.

Three additional MCO genes were found to be only present in the enzyme-producing strain CBS 513.88, confirming the significant genotypic differences between different *A. niger *strains and showing the potential of these ascomycete genomes as a source for new MCOs, that should be further investigated in order to get more insights about their possible biotechnological potential and their biological role.

## Methods

### Strains and culture media

*Escherichia coli *DH5α was used for all DNA manipulations. The *pyrA *deficient strain *A. niger *N593 was selected for the PCR amplification and homologous expression of MCO coding genes. Complete medium plates were used for spores preparation [45] and minimal medium [45], containing 50 mM of maltose as the sole carbon source and 0.1 mM of CuSO_4_, was used both in liquid and agar plate cultures for laccase production. The concentration of all nitrogen sources in the experiment for McoB synthesis optimization was 70 mM. Casamino acids used to supplement NaNO_3 _and (NH_4_)_2 _tartrate containing mediums were used in a final concentration of 0.1%.

### Molecular biology techniques and transformation

Standard methods were used for carry out DNA manipulations and *E. coli *transformations [46]. Genomic DNA was isolated from *A. niger *N593 as described previously [47]. PCR reactions were performed using proof reading Phusion polymerase (Invitrogen) according to the manufacturer's instructions. Fungal transformations were made using a similar protocol to the one described in Oliveira *et al*. [48].

### Cloning *A. niger *MCO genes

**Figure 6:**
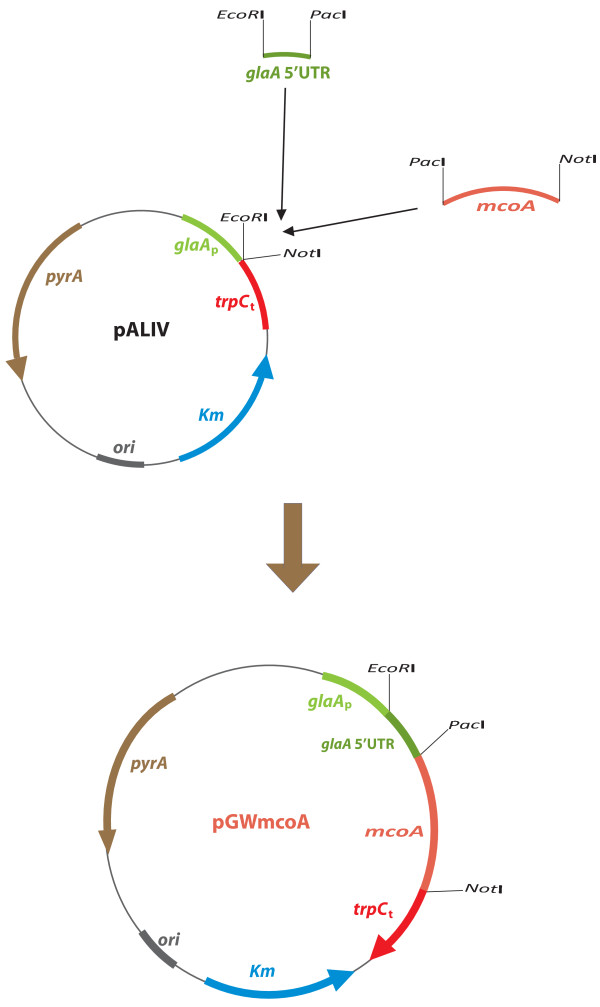
**Cloning strategy for the construction of *A. niger *expression vector pGWmcoA**.

Primers shown in Table 2 were used to amplify *A. niger *MCO genes. After their amplification, all genes were cloned in a pJET vector (Thermo Scientific) and their sequence verified. Plasmid pALIV (Figure 6), a derivative of pGW635 [49] carrying the promoter of the glucoamylase coding gene (*glaA*_p_) and the *trpC *terminator (*trpC*_t_), with *pyrA *as a selection marker, was used as an expression vector for the *A. niger *MCO genes.

**Table 2:** Primers used in this work for the amplification of the MCO coding genes in *A. niger *N593 strain

Gene	Primer	Sequence
*mcoA*	mcoA-Fw	5'-GACAACTTAATTAACCACCATGTCGCCCTTTCAATTCGGAC-3'
	
	mcoA-Rv	5'-TGTACAGCGGCCGCTCAGGAATCAGAGAGCTGGTACT-3'

*mcoB*	mcoB-Fw	5'-GACAACTTAATTAACCACCATGAGTATATCTCAGAGCAGGC-3'
	
	mcoB-Rv	5'-TGTACAGCGGCCGC CTAGATTGGCATCACTGGCAAC-3'

*mcoC*	mcoC-Fw	5'-GACAACTTAATTAACCACCATGAAGTGGTCCCATCCCAAC-3'
	
	mcoC-Rv	5'-TGTACAGCGGCCGCCTAGTCAAAGCTAGGATGATCCA-3'

*mcoD*	mcoD-Fw	5'-GACAACTTAATTAACCACCATGCACTTGCATACTATCCTGG-3'
	
	mcoD-Rv	5'-TGTACAGCGGCCGCTTAGATACCAGAATCATCCTCCTC-3'

*mcoE*	mcoE-Fw	5'-GACAACTTAATTAACCACCATGCAGCAGTCACCGTCGTTC-3'
	
	mcoE-Rv	5'-TGTACAGCGGCCGCTTACATCTGCAATAGCATGGCCA-3'

*mcoF*	mcoF-Fw	5'-GACAACTTAATTAACCACCATGTGGTTTTCTGTCTATTTCCTT-3'
	
	mcoF-Rv	5'-TGTACAGCGGCCGC TCAAACACCCGAATCGTGCTGC-3'

*mcoG*	mcoG-Fw	5'-GACAACTTAATTAACCACCATGACAATCTTTTTGCTACTCCTTGG-3'
	
	mcoG-Rv	5'-TGTACAGCGGCCGCTTATATACCCGACTCGTATGGCC-3'

*mcoI*	mcoI-Fw	5'-GACAACTTAATTAACCACCATGACTAGAACCCCACAAGTGA-3'
	
	mcoI-Rv	5'-TGTACAGCGGCCGCTTACACTCCGGAATCAATCTG-3'

*mcoJ*	mcoJ-Fw	5'-GACAACTTAATTAACCACCATGCTTTTGGAAATTTGCTGGACAG-3'
	
	mcoJ-Rv	5'-TGTACAGCGGCCGCTCAGACTCCAGAATCATCTTGGAA-3'

*mcoM*	mcoM-Fw	5'-GACAACTTAATTAACCACCATGACTCTACAAACCTACCTT-3'
	
	mcoM-Rv	5'-TGTACAGCGGCCGCTCAAATCCCCGAATCATCCTG-3'

By means of a triple-point ligation, 234 nucleotides (nt) corresponding to the *glaA *5' untranslated region (5'UTR) (synthesized by DNA 2.0 and provided in the plasmid pJref) digested with *EcoR*I-*Pac*I, and the gene *mcoA*, obtained from the pJET vector (*Pac*I-*Not*I), were ligated and placed under the control of *glaA*_p _and *trpC*_t _in *EcoR*I/*Not*I digested pALIV, generating the plasmid pGWmcoA (Figure 6). Plasmids pGWmcoB, pGWmcoC, pGWmcoD, pGWmcoE, pGWmcoF, pGWmcoG, pGWmcoI, pGWmcoJ and pGWmcoM were constructed by removing *mcoA *from pGWmcoA (*Pac*I-*Not*I) and ligating the resulting linearized plasmid with *Pac*I/*Not*I digested *mcoB*-*G*, *mcoI*, *mcoJ *and *mcoM *respectively.

**Figure 7:**
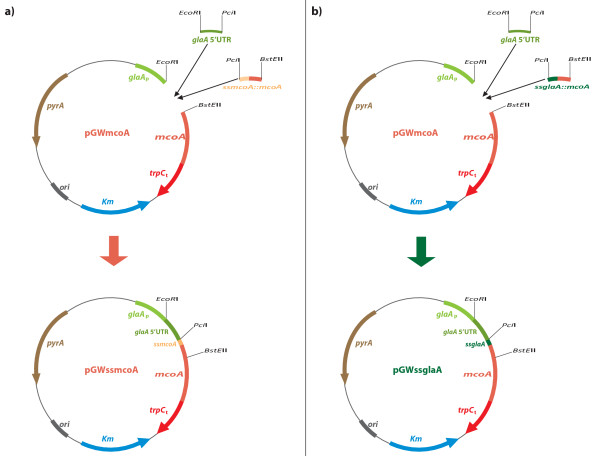
**Cloning strategy for the construction of *A. niger *expression vectors pGWssmcoA (a) and pGWssglaA (b)**.

In order to compare the McoA and GlaA signal sequences, two additional plasmids, pGWssmcoA and pGWssglaA, were constructed (Figure 7). Synthetic DNA sequences coding for the first 80 residues (240 nucleotides) of the McoA protein, including the 23 aa of its predicted signal sequence (ssMcoA), and for 78 residues (234 nucleotides) of a fusion ssGlaA::McoA, with the glucoamylase signal sequence replacing ssMcoA, were provided by DNA 2.0 in plasmids pJssMcoA and pJssGlaA respectively. The *glaA *5' UTR (202 nucleotides) was again synthesized by DNA 2.0, with different restriction sites adapted to the new cloning strategy, and provided in the plasmid pJ5ref2. Plasmid pGWmcoA was digested with *EcoR*I and *BstE*II restriction enzymes and fused in a triple point ligation with glaA 5'UTR (*EcoR*I-*Pci*I) and the first 240 nt of *mcoA *(*Pci*I-*BstE*II) to yield pGWssmcoA (Figure 7a). The plasmid pGWssglaA was generated using the same procedure, through a ligation between the linearized pGWmcoA (*EcoR*I-*BstE*II), *glaA *5'UTR (*EcoR*I-*Pci*I) and the 234 nt fragment coding for the ssGlaA::McoA fusion (*Pci*I-*BstE*II) (Figure 7b).

### Enzyme activity assays

All enzyme reactions were followed at 23°C. All chemicals were purchased from Sigma and Invitrogen. McoA activity was quantified measuring the oxidation rate of the ADBP in a reaction mixture containing 80 μL of culture supernatant and 20 μL of an ADBP/DMA solution. A 5 mM ADBP solution was prepared in 200 mM sodium acetate buffer pH 5.0 containing 25% ethanol, in the presence of 26 mM DMA. The product of the reaction between oxidized ADBP and DMA can be quantified by measuring its absorbance at 600 nm. One unit of activity was defined as the change of 1.0 in optical density at 600 nm per minute. McoB activity was quantified in 200 mM sodium acetate buffer pH 5.0 measuring the oxidation rate of 10 mM DMPPDA at 550 nm. One unit of activity was defined as the change of 1.0 in optical density at 550 nm per minute. The specific activity of the enzyme was determined after the purification of the enzyme (Tamayo-Ramos *et al*., manuscript in preparation).

The enzyme activity assays in agar plates were performed pouring a solution of either 30 mM DMPPDA, 5 mM ADBP/DMA, 2 mM DMP or 1.4 mM of ABTS in 100 mM NaAc buffer pH 5.0 over *A. niger *cultures grown at 30°C for 48 hours. The overlay solutions were discarded after an incubation time of 5 minutes.

### 96 well microcultures

Microtiter plates (1.2 ml square-well storage plates, Thermo Scientific) containing 0.8 ml of minimal medium were inoculated with 2 × 10^6 ^spores/ml, sealed with gas-permeable adhesive seals (Thermo Scientific) and incubated at 30°C and 1000 rpm in a microtron (Infors HT). After 24 hours mycelium was pelleted by centrifugation during 15 minutes at 2000 *g *and the supernatant recovered for laccase activity measurement.

### Shake-fask cultures

Erlenmeyer flasks (500 mL volume) containing 100 ml of minimal medium were inoculated with 1 × 10^6 ^spores/ml and incubated at 30°C and 250 rpm in an orbital shaker.

### Bioinformatics analysis

*A. niger in silico *genome mining was performed using the BLASTp tool (E value: 1e-3; Matrix: BLOSUM 62; Filter: Yes; Alignment type: gapped) in both the *Aspergillus *Comparative Database (strain ATCC 1015) http://www.broadinstitute.org/ (*Aspergillus *Comparative Sequencing Project, Broad Institute of Harvard and MIT) and the Central *Aspergillus *Data Repository (strain CBS 513.88) http://www.cadre-genomes.org.uk/index.html[50].

SignalP 3.0 [51] (both neural networks and Markov models methods were used), SecretomeP 2.0 (mammalian option was used) [52] and TMHMM 2.0 [53] from CBS Prediction Servers were used to predict the subcellular localization of *A. niger *MCOs. Amino acid sequences were aligned by ClustalW2 (default options provided by the program in both the pairwise alignment and the multiple sequence alignment were used) [54] from the European Bioinformatics Institute (EBI) toolbox and the phylogenetic tree was constructed with MEGA4 program [55].

## Competing interests

The authors declare that they have no competing interests.

## Authors' contributions

JATR made the bioinformatics analysis, performed the DNA cloning and microbial transformations, designed and performed the activity plate assay experiments and the data analysis and drafted the manuscript. SB designed the cloning strategy. SB and JATR designed the microculture experiment and performed the data analysis. JATR designed and performed the shake-flask culture experiment. RV collaborated in the coordination of the research. LG designed the study and helped to draft the manuscript. All authors read and approved the submission of the manuscript.

## Supplementary Material

Additional file 1 **ADBP/DMA plate assay screening for *A. niger *strains expressing *mcoJ *gene**. The conversion of the ADBP/DMA substrate can be observed by the formation of a green-blue color in the positive colonies. Different levels in color development indicate different McoJ activity levels. C+ refers to *A. niger *N593 strain expressing *A. niger *McoB as a positive control. WT refers to *A. niger *N593 transformed with the empty vector pALIV, used as a negative control. Click here for file
