# Supplementary figures and images for: The Aspergillus niger multicopper oxidase family: analysis and overexpression of laccase-like encoding genes
Source: Microb Cell Fact. 2011 Oct 8;10:78. doi: 10.1186/1475-2859-10-78 (PMC3200161; doi:10.1186/1475-2859-10-78)

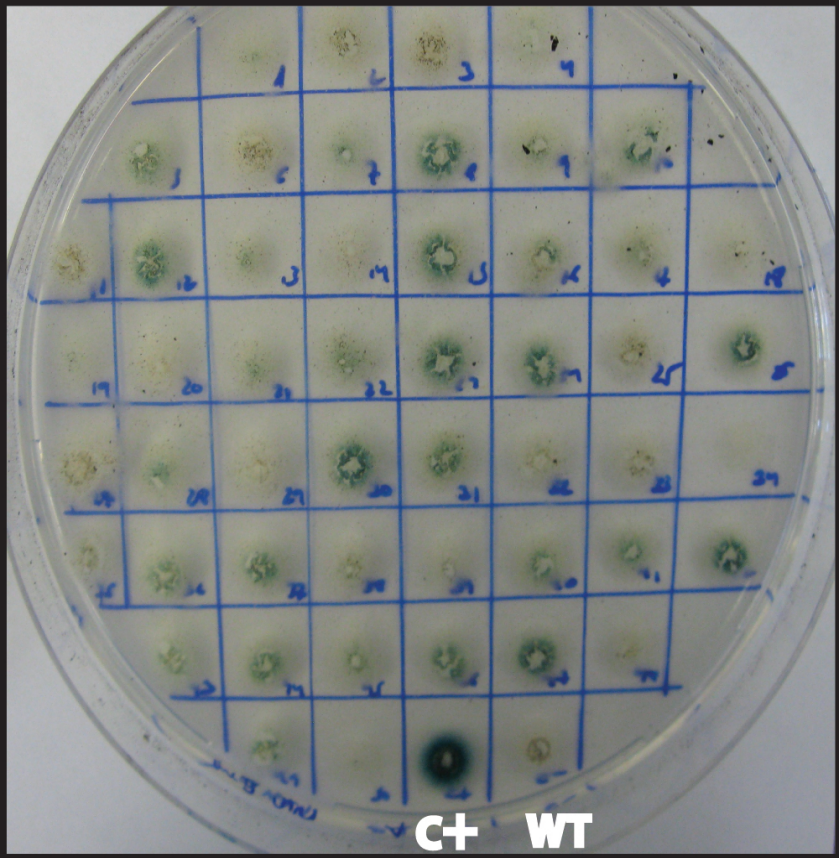

CT WT

Supplement: Additional file 1 — ADBP/DMA plate assay screening for A. niger strains expressing mcoJ gene. The conversion of the ADBP/DMA substrate can be observed by the formation of a green-blue color in the positive colonies. Different levels in color development indicate different McoJ activity levels. C+ refers to A. niger N593 strain expressing A. niger McoB as a positive control. WT refers to A. niger N593 transformed with the empty vector pALIV, used as a negative control. [file 1475-2859-10-78-S1.PDF]
